# Purification, Identification, and Inhibitory Mechanisms of a Novel ACE Inhibitory Peptide from *Torreya grandis*

**DOI:** 10.3390/nu15102374

**Published:** 2023-05-18

**Authors:** Fenghua Wu, Xiaohui Luo, Yongzhu Zhang, Peng Wang, Yinzi Chang, Zhiping He, Xingquan Liu

**Affiliations:** 1 College of Advanced Agricultural Sciences, Zhejiang Agriculture and Forestry University, Hangzhou 311300, China; 2 College of Food and Health, Zhejiang Agriculture and Forestry University, Hangzhou 311300, China; lxhcecily@yeah.net (X.L.); 20220143@zafu.edu.cn (Y.Z.); wpeng@zafu.edu.cn (P.W.); cyz12345@zafu.edu.cn (Y.C.); hzp@zafu.edu.cn (Z.H.)

**Keywords:** *Torreya grandis* meal protein, ACE inhibitory peptide, molecular docking, structure–activity relationship, inhibitory mechanism, endothelial cells

## Abstract

*Torreya grandis* meal has a high protein content and an appropriate amino acid ratio, making it an excellent protein source for producing ACE inhibitory peptides. To promote its application in food, medicine, and other fields, an alkaline protease hydrolysate of *Torreya grandis* was used in this study to isolate and identify a novel angiotensin-converting enzyme inhibitory peptide, VNDYLNW (VW-7), using ultrafiltration, gel chromatography purification, LC-MS/MS, and in silico prediction. The results show that the IC_50_ value of VW-7 was 205.98 µM. The Lineweaver–Burk plot showed that VW-7 had a mixed-type inhibitory effect on ACE. Meanwhile, according to the results of molecular docking, VW-7 demonstrated a strong affinity for ACE (binding energy −10 kcal/mol). VW-7 was bound to ACE through multiple binding sites. In addition, VW-7 could remain active during gastrointestinal digestion in vitro. Nitric oxide (NO) generation in human endothelial cells could rise after receiving a pretreatment with VW-7. These results indicated that *Torreya grandis* meal protein can be developed into products with antihypertensive function, and VW-7 has broad application prospects in the field of antihypertensive.

## 1. Introduction

*Torreya grandis* (*T. grandis*) is a special monetary tree species with lengthy cultivation records in China [1]. The fruit has a rich dietary value and contains rich amounts of protein, unsaturated fatty acids, vitamins, and minerals [2,3]. It has the ability to improve human immunity and prevent cardiovascular diseases [4]. In recent years, with the growing area of *Torreya grandis*, enterprises that specialize in this species have gradually shifted from selling nuts to developing refined *Torreya grandis* products. *T. grandis* is also rich in valuable oil, and the process of extracting this resource creates a lot of protein-rich processing waste [5]. The utilization of this resource presents a significant challenge that has yet to be solved.

Previous studies have found that the protein content of *T. grandis* seeds varied from 10.34% to 16.43% depending on the cultivar [6], and the protein content of meal increased after oil extraction. High-quality protein resources are the raw materials of functionally active peptides. Antioxidant activity in *T. grandis* protein hydrolysates has previously been documented [7], and we have also conducted a number of studies on *T. grandis* antioxidant peptides [8]. Previous studies have shown that foods or proteins with high levels of valine (Val), arginine (Arg), leucine (Leu), proline (Pro), histidine (His), phenylalanine (Phe), threonine (Thr), methionine (Met), tyrosine (Tyr), and lysine (Lys) are suitable for the preparation of ACE inhibitory peptides [9,10]. According to the previous laboratory study in *T. grandis*, the total amount of the above amino acids accounted for about 50.34% [8], which suggested that *T. grandis* might be an appropriate raw material for the production of ACE-inhibiting peptide. However, no trustworthy research has been done to discover and assess the ACE inhibitory peptide of *T. grandis*.

Hypertension is one of the most prevalent chronic conditions that can result in cardiovascular and cerebrovascular disorders and is a serious health issue affecting people all over the world [11]. An efficient target for controlling blood pressure is thought to be the zinc dipeptide-carboxypeptidase known as angiotensin-converting enzyme (ACE). It can cleave the inactive angiotensin I in the renin-angiotensin system (RAS) to form the potent vasopressor angiotensin II [12]. Furthermore, ACE also inactivates the vasodilator Bradykinin in the kinin kinase system (KKS), leading to an increase in blood pressure [13]. Therefore, inhibition or inactivation of ACE is recognized as a straightforward approach to relieving hypertension [14]. Vasodilation is one of the main mechanisms of antihypertensive effects [15]. Bradykinin is an effective mediator of endothelium-dependent vasodilation and regulates various biological processes involved in the stimulation of endothelial nitric oxide (NO) synthesis [16]. NO is thought to be the most significant endothelial cell-derived factor with vasodilatory properties. By binding to B2 receptors on the surface of endothelial cells, bradykinin, which is increased by ACE inhibitors, activates eNOS and boosts NO generation, ultimately regulating vasodilation and lowering blood pressure [16]. Many researchers have investigated how ACE inhibitory peptides affect cells, such as changes in cell NO production [17], ACE activity [18], and gene expression [19].

In this study, *T. grandis* meal protein was hydrolyzed with the aid of alkaline protease, and the hydrolysate was isolated and purified using ultrafiltration and gel filtration chromatography (GFC) to obtain ACE inhibitory peptides. The sequences of GFC-derived peptides had been determined by LC-MS/MS. Peptide sequences were screened by molecular docking combined with the Peptide Ranker database, and then the selected peptides were synthesized to verify whether they had ACE inhibitory activity. The peptide with the strongest ACE inhibitory activity was identified, and its inhibition mode was explored by the Lineweaver–Burk plot. In addition, the gastrointestinal stability of the peptide with the strongest ACE inhibitory activity was also determined. The possible mechanisms of ACE inhibitory activity were also investigated, and preliminary studies were conducted to investigate the effect of peptides on hypertension-related NO expression at the cellular level. The results derived from this study may provide available information for *T. grandis* meal proteins and elucidate the possible inhibition mechanism of ACE inhibitory peptides.

## 2. Materials and Methods

### 2.1. Materials and Chemicals

The *T. grandis* was purchased from a local market in Fuyang County, Hangzhou City, Zhejiang Province, China. EA.hy926 cells were kindly provided by the Cell Bank, Chinese Academy of Sciences. Alkaline protease (EC 3.4.21.14, 200 U/mg) and Sephadex G-15 were acquired from Beijing Solarbio Biotechnology Co., Ltd. (Beijing, China). ACE (EC 3.4.15.1) and *N*-Benzoyl-Gly-His-Leu (HHL) were obtained from Sigma- Aldrich Co., Ltd. (St. Louis, MO, USA). Cell Counting Kit-8 (CCK-8) and NO assay kits were purchased from Beyotime Biotechnology Co., Ltd. (Shanghai, China). All other reagents were of analytical grade.

### 2.2. Methods

#### 2.2.1. Preparation of *T. grandis* Meal Protein Hydrolysate (TGMPH)

*T. grandis* protein (2%, *w*/*v*) was dispersed in pure water and denatured at 95 °C for 15 min. The solution was adjusted to pH 10 after cooling, and 5000 U/g alkaline protease was added. The reaction was stirred at 50 ℃ for 4 h. Immediately after the reaction, the enzyme was deactivated at 95 °C for 15 min. Finally, after centrifuging the supernatant at 8500 rpm for 30 min at 4 °C, it was lyophilized and labeled as TGMPH for the subsequent experiments.

#### 2.2.2. Purification of ACE Inhibitory Peptides

TGMPH (T0) was successively filtered using an ultrafiltration apparatus (Millipore, Bedford, MA, USA). Four fractions, T1 > 10 kDa, T2 = 3–10 kDa, T3 = 1–3 kDa and T4 < 1 kDa were obtained. Each fraction was concentrated, lyophilized, and frozen at −20 ℃ for later use.

The strongest ACE inhibitory fraction obtained by ultrafiltration was dissolved in deionized water (10 mg/mL) and separated using a Sephadex G-15 gel filtration column (1.6 cm × 70 cm). Three milliliters of the sample solution was placed onto a well-balanced gel column at a flow rate of 0.6 mL/min for elution after the column had been pre-equilibrated with distilled water. An automatic partial collector was used to collect fractions at a rate of two min/tube, and the sample’s absorbance was measured at 280 nm. For subsequent usage, each fraction was concentrated, lyophilized, and kept at −20 ℃.

#### 2.2.3. Measurement of ACE Inhibitory Activity

With a minor modification, Sutopo et al.’s [14] methodologies were used for the ACE inhibitory activity assay. For each assay, 60 µL of substrate (2.5 mM HHL, prepared with borax and boric acid buffer of pH 8.3) was mixed with 20 µL of sample solution (diluted with borax and boric acid buffer of pH 8.3), and pre-incubated for 5 min at 37 °C before being incubated for 1 h with 40 µL of ACE solution (0.05 U/mL) at 37 °C. Afterwards, 120 µL of HCl (1 M) was added to terminate the reaction. The solution was filtered through a 0.45 µm stream filtration membrane for HPLC analysis (Shimadzu LC-20AT) with a column C18 Inertsil ODS-SP (4.6 mm × 250 mm, 5 µm). The hydrolyzed product of HHL, HA, was quantified by its chromatographic peak area. Acetonitrile/ultrapure water solution (1:3, each containing 0.1% trifluoroacetic acid) was processed with isocratic elution at a constant flow rate of 1.0 mL/min, the wavelength of the UV detector was set at 228 nm; the column temperature used was 30 ℃; and the injection volume quantity used was 10 µL. The inhibitory activity of ACE was calculated according to Equation (1):

(1) $$\mathrm{ACE}\;\mathrm{inhibition}\;\mathrm{rate}\%=\frac{\left(A0\;-\;A\right)}{A0}\;\times \;100\;$$

where A0 is the hippuric acid peak area of the blank sample (the sample is replaced by buffer solution), and A is the hippuric acid peak area of the sample.

#### 2.2.4. Identification of ACE Inhibitory Peptides

The Easy-nLC 1200 system was used in conjunction with a Q Exactive™ Hybrid Quadrupole-Orbitrap™ (Thermo Fisher Scientific, Waltham, MA, USA) equipped with an ESI nanospray source to determine the amino acid sequences of the peptide purified by GFC that showed high ACE inhibitory action. The sample was reduced, alkylated, desalted, and loaded onto the C18 precolumn (300 µm × 5 mm, 5 µm) followed by a C18 nano-analytical column (150 µm × 150 mm, 1.9 µm). A two-solvent system was used: (A) 0.1% formic acid in water and (B) 20% 0.1% formic acid in water and 80% acetonitrile. The gradient elution was conducted with a gradient of B: 0–2 min, 4–8%; 2–45 min, 8–28%; 45–55 min, 28–40%; 55–56 min, 40–95%; 56–66 min, 95%, with a flow rate of 600 nL/min. A MS/MS full-scan was performed for the sample with a 100–1500 mass/charge (m/z) acquisition range in MS mode. MS/MS conditions were as follows: Activation Type, HCD; Normalized Coll. Energy, 8.0; Activation Time, 66.0 min. From the Orbitrap preview scan, the twenty most intense peptide ions were chosen as the foundation for peptide identification. The LC-MS/MS data were analyzed using PEAKS Studio 8.5, and only the peptides with high levels of confidence were selected for further protein identification research.

#### 2.2.5. In Silico Prediction of ACE Inhibitory Peptides 

Different bioactivity prediction servers were used to predict the ACE inhibitory peptide sequences obtained from the LC-MS/MS analysis of the selected samples. The PeptideRanker website (http://distilldeep.ucd.ie/PeptideRanker/ (accessed on 24 May 2022)) was used to predict the potential biological activity [20]. The peptides with active scores > 0.5 had been identified as promising bioactive peptides. The ToxinPred website (http://www.imtech.res.in/raghava/toxinpred/ (accessed on 24 May 2022)) was used to predict the selected peptides toxicity [21].

#### 2.2.6. Molecular Docking

Using the Autodock Vina software, molecular docking was performed on the identified peptides. The PDB website (http://www.rcsb.org (accessed on 28 May 2022)) provided the download link for the ACE crystal structure (PDB ID: 1O8A). The Autodock Tool 1.5.6 software was used to process it, including deleting water molecules, adding polar hydrogen atoms and an electric field, and adding a 0.95e positive charge of Zn^2+^. The structures of peptides were generated by ChemOffice 2018. When docking, the center coordinates of the search space were x = 43.821, y = 38.24, and z = 46.712, and the search space ranges x, y, and z were all 100. The docking result was expressed as the binding energy value.

#### 2.2.7. Peptide Synthesis

The new peptides discovered through in silico screening were synthesized by Nanjing Jinsirui Biotechnology Co., Ltd. (Nanjing, China) using the solid phase synthesis method in order to evaluate their in vitro ACE inhibitory efficacy. Synthetic peptides with a purity greater than 95% were used in this study.

#### 2.2.8. Inhibitory Kinetics Study

Feng et al. [22] approach was used to examine the active peptides’ ACE inhibitory kinetics. Various HHL concentrations (2, 3, 4, and 5 mM) and peptide concentrations (0, 0.1, and 0.4 mg/mL) were incubated with the ACE solution. One unit of ACE activity was defined as the conversion of 1 μmol HHL to HA within 1 min at pH 8.3 and 37 ℃. The peak area obtained by HPLC was substituted into the standard curve of HA: y = 28,628x + 17,135, and the product concentration (C) was calculated. The product formation rate (V) was calculated according to Equation (2):

(2) $$V=\frac{C}{\Delta t}$$

where Δt is the reaction time: 60 min.

A Lineweaver–Burk plot was used to analyze the inhibitory pattern, which was based on the reciprocal of the product formation rate (1/V) and the HHL concentration (1/[S]). The kinetic constants are calculated by the master diagram, where the x-axis intercept is 1/Km and the y-axis intercept is 1/Vmax. In addition, the inhibition constant (Ki) was obtained from a secondary plot of the slope of the Lineweaver–Burk line versus peptide concentration, and the x-axis intercept of the linear graph was −Ki.

#### 2.2.9. Stability of VW-7 against Simulated Gastrointestinal Digestion

The simulated gastrointestinal digestion method was applied according to Li et al. [23], with some modifications. In brief, pH 2.0 was adjusted for the VW-7 solution (ultrapure water, c = 0.4 mg/mL). The peptide solution was mixed with pepsin (2%, *w*/*w*) for 2 h at 37 °C. The pH was changed to 7.0 after the process, and half of the liquid was withdrawn. The other half was then given trypsin (2%, *w*/*w*) and digested at 37 °C for 2 h. Finally, the reaction was terminated by a boiling water bath, and the ACE inhibition rate of the two-step reaction products was measured.

RP-HPLC was used to analyze the liquid obtained after digestion. The mobile phase A was water containing 0.1% TFA, and the mobile phase B was 100% ACN containing 0.1% TFA. The flow rate was set to 1 mL/min, the detection wavelength was 214 nm, and the column temperature was 30 °C. The specific elution procedure is shown in Table 1.

#### 2.2.10. Cell Culture

Human umbilical vein endothelial cells (EA.hy926) were grown in DMEM with 10% FBS and 1% penicillin-streptomycin at 37 °C in a humidified environment of 5% CO_2_. 

#### 2.2.11. Cell Viability Determination

Using a cell counting kit-8 (CCK-8) kit, the cytotoxicity of the peptide VNDYLNW in EA.hy926 was assessed. In 96-well cell culture plates, cells were inoculated at a density of 5 × 10^3^/well. After 24 h of pre-incubation at 37 ℃ and 5% CO_2_, 50 µL of peptides with different concentrations were added to achieve the final concentrations of solution of 100, 200, 400, 600, 800, and 1000 µg/mL (using complete medium as the control) for 24 h. Following treatment, the culture medium was removed, 100 µL of medium (without FBS) was added, and 10 µL of CCK-8 was then added to each well. The culture was incubated for 1 h at 37 ℃ and 5% CO_2_. Finally, the light absorption value at 450 nm was detected by the microplate reader. Relative cell viability was calculated according to Equation (3):

(3) $$\mathrm{Cell}\;\mathrm{viability}\%=\frac{\left(A\;-\;A0\right)}{A1\;-\;A0}\;\times \;100\;$$

where A1 is the absorbance of cells that are not treated with peptides, A is the absorbance of cells that are treated with peptides, and A0 is the absorbance of the group without cells in the medium.

#### 2.2.12. Measurement of NO Levels

NO generation was measured using the NO fluorescent probe (DAF-FM DA) [11]. Cells (6 × 10^4^ cells/mL) were cultured in 12-well plates and cultured in a humid environment of 37 ℃ and 5% CO_2_ for 24 h. 250 µL of peptide of different concentrations were added to achieve the final concentrations of solution, which were 0, 100, 200, and 400 µg/mL for 24 h. After the culture process, the culture medium was discarded, and pancreatic enzymes were used to digest and collect cells. Afterward, the DAF-FM-DA solution (5 µM) was added, and it was incubated at 37 °C in the dark for 20 min. A fluorescent microplate reader was used to measure fluorescence after incubation. 

#### 2.2.13. Statistical Analysis 

The data are reported as mean ± SD and each experiment was repeated at least three times. SPSS Statistics 26 was used for analysis, and Duncan’s model was used to analyze the significant differences between different samples, where *p* < 0.05 means a significant difference and using different lowercase letters means a different level of significance.

## 3. Results and Discussion 

### 3.1. Isolation of ACE Inhibitory Peptides from TGMPH

The protein hydrolysate obtained after enzymolysis is a mixture composed of residual protein, peptides, and free amino acids. It is required to separate and purify the protein hydrolysate in order to investigate the structure–activity relationship of active peptides, elucidate the inhibition mechanism, and obtain high-purity peptides. According to the difference in molecular weight (MW), charge, and hydrophilicity of the mixture, different methods can be used for separation and purification. At present, the commonly used methods are ultrafiltration, gel chromatography, RP-HPLC, and so on.

**Figure 1:**
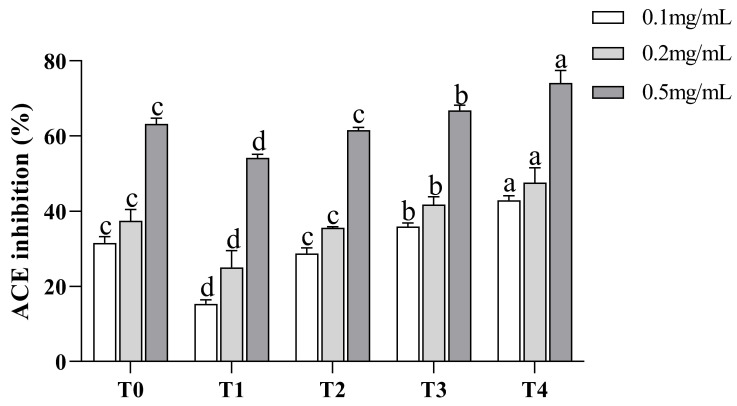
ACE inhibitory activity of *Torreya grandis* protein hydrolysate and its four ultrafiltration fractions at concentrations of 0.1 mg/mL, 0.2 mg/mL, and 0.5 mg/mL. The sample were abbreviated as follows: protein hydrolysate (T0), MW > 10 kDa (T1), MW = 3–10 kDa (T2), MW = 1–3 kDa (T3), and MW < 1 kDa (T4). Different letters above the column indicate a significant difference (*p* < 0.05).

For the isolation of peptides with various MWs, ultrafiltration was frequently utilized. [24]. In our study, TGMPH (T0) was separated into four fractions by ultrafiltration, including T1, T2, T3, and T4. Analysis was done on each fraction’s ACE inhibitory activity (Figure 1). The results showed that the activity of all peptide components was positively correlated with their concentration. With the increase in concentration, the ACE inhibition ability of each component was significantly enhanced (*p* < 0.05). In addition, the fractions with different MW had different ACE inhibitory activities, in the following order: T4 > T3 > T2 > TGMPH (T0) > T1. When the concentration reached 0.5 mg/mL, the scavenging activity of each fraction was greater than 50%. It indicated that T4 made a significant contribution to the ACE inhibitory activity of T0 (*p* < 0.05), and this fraction was selected for further purification. These results are consistent with the literature findings that fractions with lower MW have stronger ACE inhibitory activity. Sompinit et al. [25] found in their study of ginger that the <1 kDa fraction had the strongest ACE inhibitory activity. Previous studies have also shown that the activity of peptides is related to their MW. Having a low MW is characteristic of most of the identified ACE inhibitory peptides [24], which may be due to the inability of the ACE active site to accommodate long peptide sequences [26]. 

**Figure 2:**
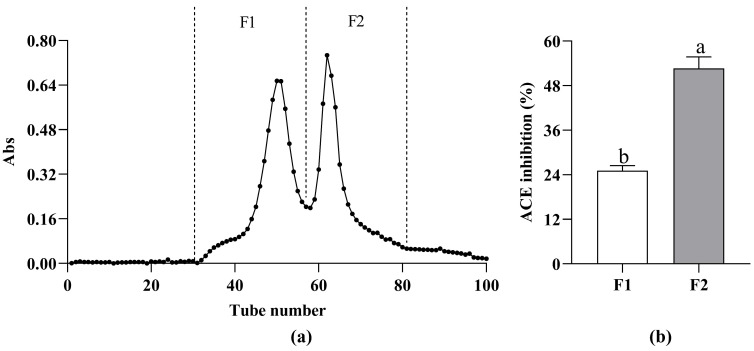
Elution profile of fractions T4 separated with Sephadex G-15 gel chromatography (**a**) and ACE inhibitory activity of fractions F1 and F2 (c = 0.2 mg/mL) (**b**). Different letters above the column indicate a significant difference (*p* < 0.05).

Gel filtration chromatography is based on the hydrodynamic radius or molecular dimension for separation and purification [17]. Several ranges of peptides can be separated according to the different gels used in the experiment. Because of the MW of T4 < 1 kDa, the Sephadex G-15 was selected for purification. T4 was separated into two chromatographic peaks (Figure 2a). Both fractions showed ACE inhibitory activity; however, at a concentration of 0.2 mg/mL (Figure 2b), F1’s ACE inhibitory activity was 25.08 ± 1.41%, which was significantly lower than that of F2 (52.60 ± 3.12%). Therefore, F2 was considered to be the most effective part of T0 in ACE inhibitory activity, and this fraction was selected for subsequent analysis.

### 3.2. Identification and Screening of the Active ACE Inhibitory Peptides

For peptide identification, the peptide mixture of GFC fraction F2 was injected into LC-MS/MS. PEAKS Studio8.5 was used to analyze the raw MS files, and 359 peptides were identified. The traditional bioactive peptide screening method is time-consuming and laborious [27]. Hence, bioinformatics is used to replace traditional methods for rapid screening of peptides. 

Firstly, 37 peptides with peak areas ≥ 10^8^ and PEAKS Studio scores ≥ 95 were selected for analysis. To determine the most effective ACE inhibitory peptide from the 37 peptides, in silico analyses were performed using molecular docking and the database PeptideRanker.

Molecular docking is an intuitive and effective technique to learn about the interplay between small molecular ligands and receptors [28]. The binding energy required for ligand binding to receptors can be used as a significant criterion for ligand screening; the higher the relative value, the closer the peptide binds to ACE [29,30]. Table 2 displays the results of the binding energies between ACE and peptides, with 25 peptides having binding energies less than or equal to −8.5 kcal/mol. Xie et al. [29] screened eight peptides with binding energy < −10 kcal/mol in α-lactalbumin hydrolysates to verify ACE inhibitory activity, among which 5 peptides had good ACE inhibitory activity, and their IC_50_ values were all lower than 18.0 µM, indicating that molecular docking screening had high accuracy.

In the silicon analysis based on the PeptideRanker database, in order to reduce false negatives and obtain as many potential bioactive peptides as possible, we set the score screening threshold to 0.5; that is, peptides with a score higher than 0.5 are considered to have potential biological activity [27]. A total of 17 peptides meet the requirements. Gao et al. [31] found that 8 peptides with a PeptideRanker score > 0.5 in milk protein hydrolysates had prominent xanthine oxidase inhibitory activity, indicating that PeptideRanker is a powerful tool to improve the screening efficiency of bioactive peptides. In addition, ToxinPred was used to conduct yet another in silico analysis in order to calculate the toxicity of these peptides. The results showed that 37 peptides were non-toxic, indicating that they could be used in the fields of health, food, or medicine. After comprehensive consideration, 11 peptides with binding energies less than −8.5 kcal/mol and PeptideRanker scores greater than 0.5 were selected for synthesis verification. The peptides were as follows: AYRDF, VNDYLNW, FFFN, FFNVN, FDKLLSPR, FFDLK, NRNPPSLL, VVPF, FFDK, WMLV, and GYGL.

### 3.3. ACE Inhibitory Activity of Synthesized Peptide

**Figure 3:**
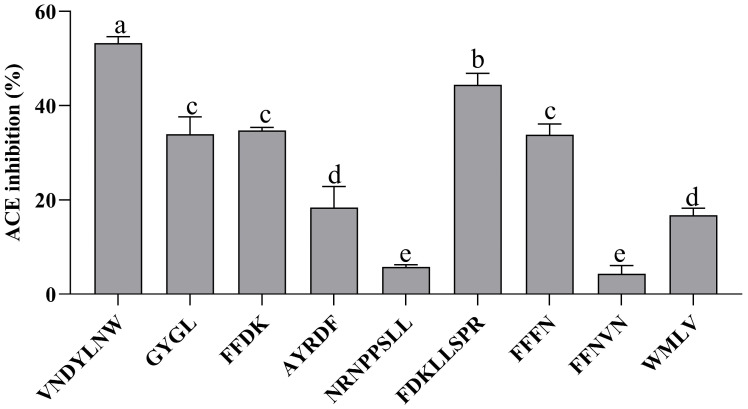
ACE inhibitory activity of the synthesized peptides identified from fraction F2 at a concentration of 0.2 mg/mL. Different letters above the column indicate a significant difference (*p* < 0.05).

**Figure 4:**
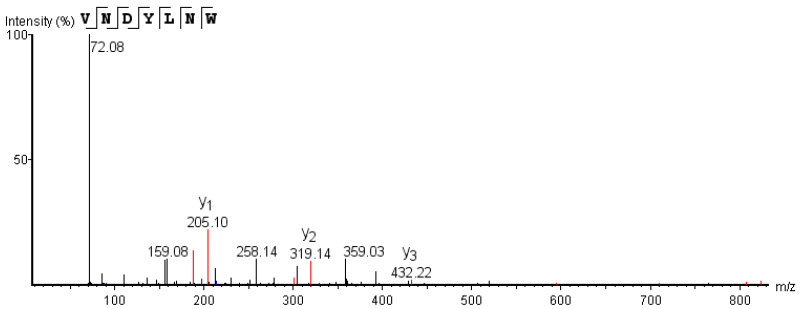
ESI-MS/MS spectrum of VNDYLNW identified in *Torreya grandis* protein-isolated hydrolysate.

As shown in Figure 3, each synthetic peptide was prepared at 0.2 mg/mL, and the ACE inhibitory activity of each sample was determined. The remaining 9 peptides, with the exception of VVPF and FFDLK, had varying degrees of ACE inhibitory action, demonstrating the success of the in silico screening. VNDYLNW showed the highest ACE inhibitory activity (53.27 ± 1.38%), followed by FDKLLSPR (44.40 ± 2.45%), FFDK (34.71 ± 0.66%), GYGL (33.94 ± 3.70%), and FFFN (33.81 ± 2.33%). The ACE inhibitory activity of the remaining peptides was low, and the inhibition rate was below 20%. Here, we measured the IC_50_ of VNDYLNW (VW-7), which is 205.98 µM, and its secondary mass spectrometry is shown in Figure 4. Chen et al. [32] reported that peptides having IC_50_ values between 0.32 and 1000 µM were deemed to have the potential to lower blood pressure. Compared with other reported ACE-inhibiting peptides, the ACE inhibitory activity of polypeptide VW-7 was stronger than that of VDMF (IC_50_: 382.28 µM) extracted from Coix prolamin [33], but weaker than that of VTPVGVPKW (IC_50_: 1.8 µM) extracted from Black Cumin seeds [14]. 

The features of ACE inhibitory peptides have been outlined in various studies. For example, the activity of ACE inhibitory peptides is affected by the last three amino acid residues at the *C*-terminal of the peptide [34]. These three positions of strong active ACE inhibitory peptides are more likely to contain hydrophobic, aromatic, or branched side chain amino acid residues. At the last location of the *C*-terminal end of the peptide, Tyr (Y), Phe (F), Trp (W), or Pro (P) are preferable [34,35]. The hydrophobic *N*-terminal is another common structural feature of ACE inhibitory peptides. Leu plays an important role in improving the activity of ACE inhibitors. In addition, hydrophobic amino acids may make the peptides have a higher affinity for the active site of ACE [36]. In summary, combined with the peptide obtained in this experiment, it can be found that the *N*-terminal of VW-7 is a hydrophobic amino acid, Val. The last three amino acids at the *C*-terminal contain two hydrophobic amino acids, Leu and Trp, and the last one at the *C*-terminal is Trp, which may give VW-7 higher ACE inhibitory activity. However, these rules may not be universal; for example, in this study, VVPF conformed to most of the above rules, but no ACE inhibitory activity was detected. Conversely, GYGL, which hardly meets the rules, shows strong activity. The difference in the results may be due to the fact that the amino acid composition may not fully determine the ACE inhibitory activity of the peptide; and that the activity may be affected by other factors such as spatial conformation and hydrophobicity [27]. Because VW-7 has the strongest ACE inhibitory activity, it was chosen for further study.

### 3.4. Binding Sites and Force Analysis of ACE and Peptides

Molecular docking is one of the key tools in structural molecular biology. In this study, we explored the interaction between peptide and ACE to further elucidate the mechanism of ACE inhibition activity. ACE contains three major active pockets, including S1 (Ala354, Glu384, Tyr523), S1’ (Glu162), and S2 (Gln281, His353, Lys511, His513, Tyr 520) [37]. In addition, ACE is a zinc-dependent dipeptide carboxypeptidase [38]. Zn^2+^ is also the active site of ACE and plays a crucial role in ACE inhibitory activity. Meanwhile, the tetrahedral ligand that Zn^2+^ forms with His387, His383, and Glu411 is crucial to the binding affinity between ACE and inhibitors [30,39]. This active site region is also known as the HEXXH zinc-binding motif [27]. Many ACE inhibitors, including captopril and enalapril, can interact with Zn^2+^ [40]. Previous studies have shown that hydrogen bonding is the major force affecting the specificity and stability of peptide-receptor complexes [32].

**Figure 5:**
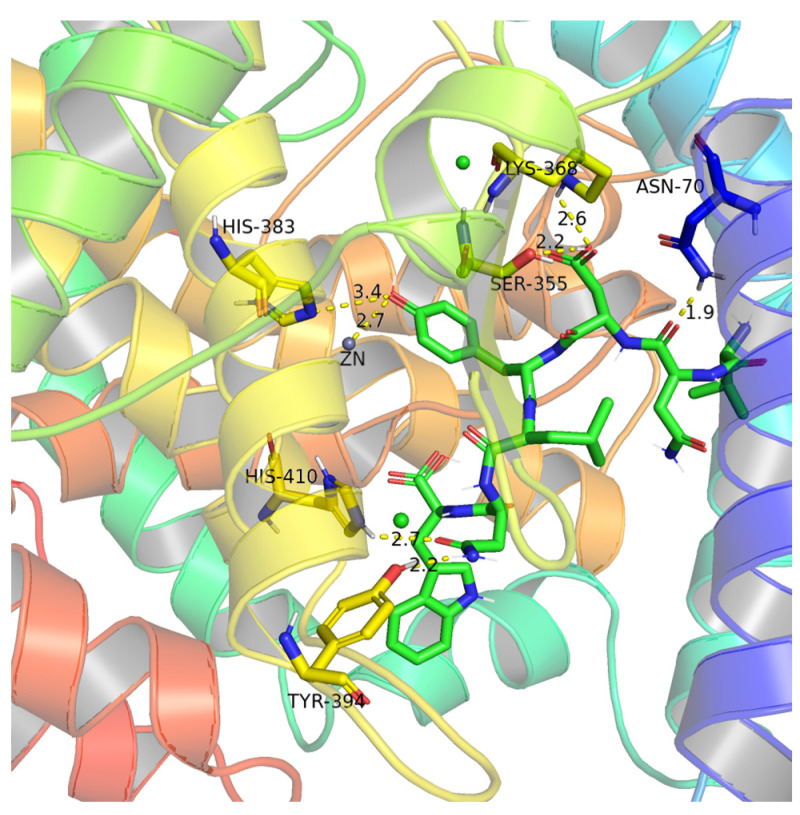
Molecular interaction between inhibitory peptide VNDYLNW and ACE (PDB: 1O8A).

As shown in Figure 5, VW-7 was found to form hydrogen bonds with six amino acid residues of ACE: Asn70, Lys368, Ser355, His410, Tyr394, and His383, but except for His383 at the HEXXH zinc-binding motif, the remaining amino acid residues are not in the active pocket. In addition, VW-7 can also be bonded directly to Zn^2+^ by a hydrogen bond. Previous studies have suggested that the interaction between the ACE inhibitory peptide and Zn^2+^ may lead to structural changes of the active site [17]. It has also been proposed that effective binding to the Zn^2+^ active site may cause the peptide to show competitive inhibition [32]. Moreover, VW-7 can also form hydrogen bonds with Zn^2+^-coordinated residue (His383), which may result in the deformation of the tetrahedral structure of the Zn^2+^ complex, leading to ACE inactivation [39].

### 3.5. ACE Inhibition Mechanism of the Synthetic Peptides

**Figure 6:**
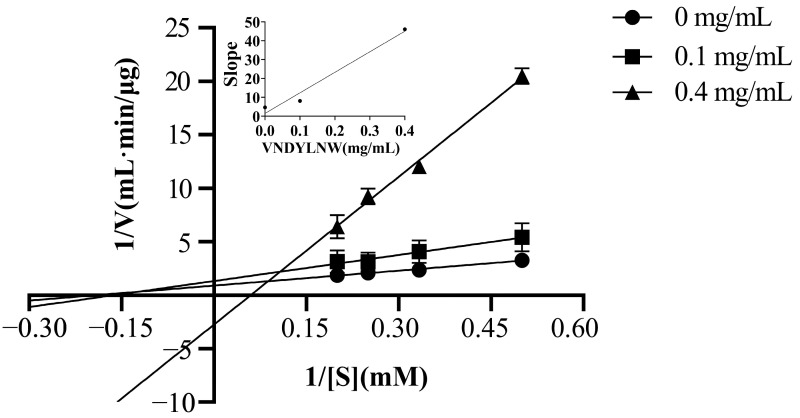
Lineweaver–Burk plot and inhibition constant quadratic plot of the peptide VNDYLNW inhibiting ACE. [1/S] and [1/V] represent the reciprocals of substrate concentration and reaction velocity, respectively.

Based on the Lineweaver–Burk plot analysis, the inhibitory mechanism of VW-7 on ACE was investigated. According to Table 3, the Vmax value decreased and the Km value increased as the peptide concentration increased. Additionally, these three curves did not intersect either the x axis or the y axis (Figure 6). This indicated that the peptide inhibitor VW-7 was acting as a mixed-type inhibitor [41]. The mixed inhibition indicated that VW-7 could bind both the active site and the allosteric site of ACE [42], which was consistent with the results of molecular docking. When the peptide binds to the active site of the enzyme, it would prevent substrate binding; however, when the inhibitor binds to the inactive site, it would lead to a conformational distortion of the enzyme protein, thereby reducing ACE substrate interaction [17]. In recent years, many ACE inhibitory peptides with mixed-type inhibition have been reported, such as EVSQGRP, VSRHFASYAN, and SAAVGSP from sea cucumber [43], NMAINPSKENLCSTFCK from casein [41], lentinus edodes polypeptide, agaric polypeptide [44], etc.

It is reported that Ki was used to measure the binding strength between inhibitors and ACE. The higher the affinity, the lower the Ki value [45]. The inhibition constant Ki of VNDYLNW on ACE was 13.61 μM, which was lower than that of α-lactalbumin peptide [46] and higher than that of the corn gluten peptide [45].

### 3.6. Gastrointestinal Digestion Stability of VW-7

**Figure 7:**
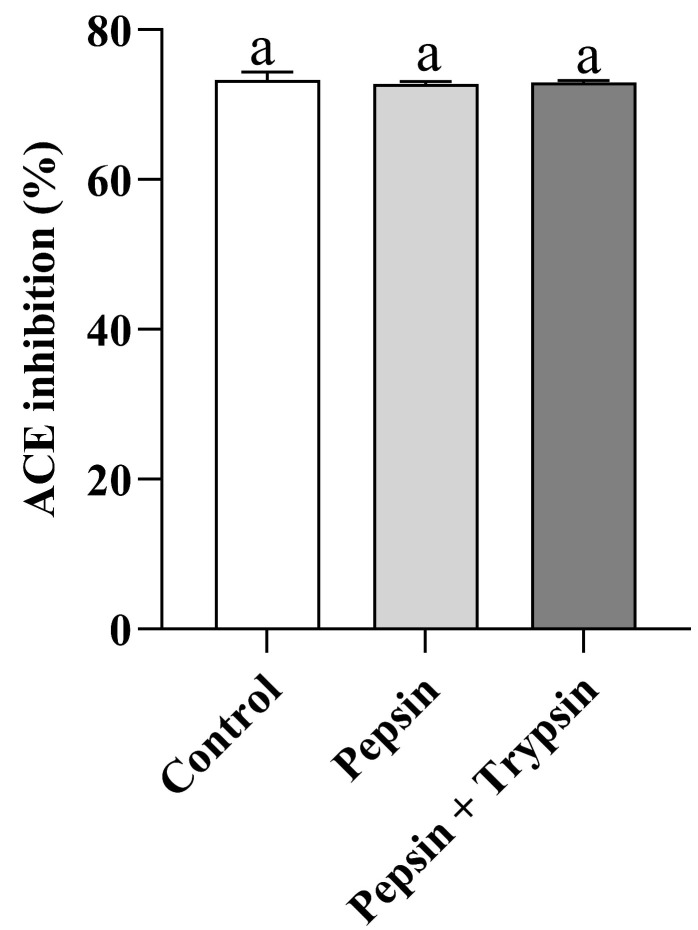
Effects of gastrointestinal digestion on the ACE inhibitory activity of VNDYLNW. Different letters above the column indicate a significant difference (*p* < 0.05).

**Figure 8:**
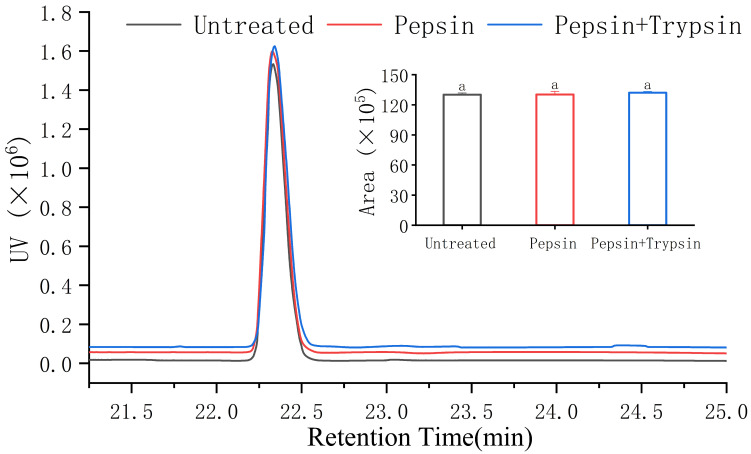
RP-HPLC chromatogram of VNDYLNW after gastrointestinal digestion treatments. Different letters above the column indicate a significant difference (*p* < 0.05).

To apply their activity in the body, peptides must be able to withstand gastrointestinal digestion or breakdown, regardless of whether they are used as functional food ingredients, food additives, or in medicine research [47]. Due to the high cost of animal experiments and clinical trials, the use of proteases for in vitro simulated digestion experiments has become a popular method for studying the stability of gastrointestinal digestion [48]. Therefore, we looked at VW-7’s resistance to gastrointestinal digestion (Figure 7). After being broken down by trypsin and pepsin, it was discovered that the ACE inhibitory action of VW-7 remained essentially unaltered (*p* > 0.05). These findings suggest that VW-7 remained stable throughout the simulated digestive process. Previous studies have suggested that stable peptides are usually composed of branched-chain amino acids and aromatic amino acids, including Leu, Ile, Tyr, and Val [49], which may be one of the reasons for the high digestive stability of VW-7. In order to further determine whether the peptides changed after digestion, we simulated gastrointestinal digestion, and used RP-HPLC to evaluate the peptides. It can be seen from Figure 8 that the peak area of VW-7 did not change after two-step digestion, indicating that the peptide structure did not change and had good stability against gastrointestinal digestion. This result indicated that VNDYLNW has the potential to become an antihypertensive drug in vivo. 

### 3.7. Cell Viability Assay 

**Figure 9:**
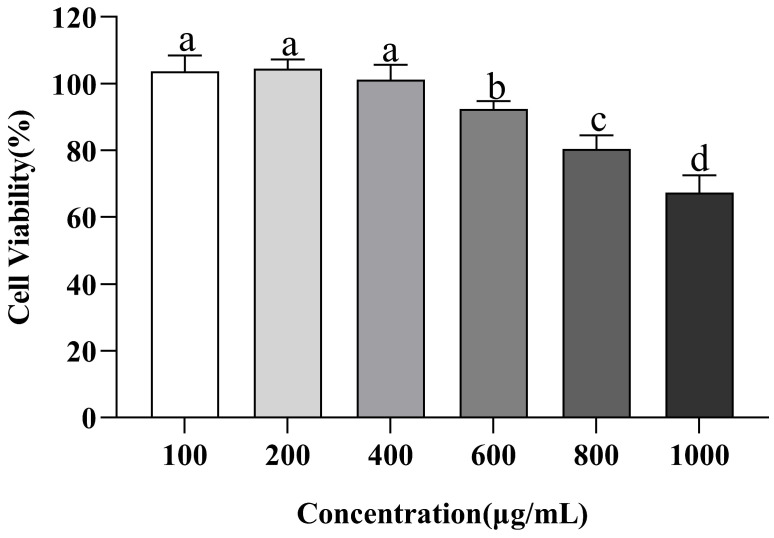
Effect of VNDYLNW on cell viability of EA.hy926 cells. Different letters above the column indicate a significant difference (*p* < 0.05).

Currently, many foodborne ACE inhibitory peptides have been discovered. Whether these peptides cause an immune response in humans and are metabolized into poisonous byproducts needs further investigation [50]. In normal tissues, cell proliferation and apoptosis maintain a dynamic balance. Previous studies on the cellular level of peptides have found that peptides that show strong proliferation inhibition in normal cells are not suitable for the development of products that are not anti-tumor functional because they are thought to pose potential risks to the integrity of tissues and organs [51]. In this study, the effect of VW-7 on the cell activity of EA.hy926 was evaluated using the CCK-8 method at multiple concentrations of VW-7 (0–1000 µg/mL) (Figure 9). With the increase in peptide concentration, the cell viability of EA.hy926 cells revealed a tendency to initially remain steady before gradually declining. In the concentration range of 0–400 μg/mL, the peptide VNDYLNW had no toxic effect on EA.hy926 cells. When the concentration was 600 µg/mL, the peptide began to have an effect on cells, and the cell viability was 92.37 ± 2.40%. With a continued increase in peptide concentration, cell viability rapidly decreased to below 80%, and the toxic effect of peptides on cells was significantly enhanced (*p* < 0.05). According to the results of the cell viability test, the concentrations of ACE inhibitory peptide in the low-dose, medium-dose, and high-dose groups were 100 µg/mL, 200 µg/mL, and 400 µg/mL, respectively.

### 3.8. Evaluation of NO Production

**Figure 10:**
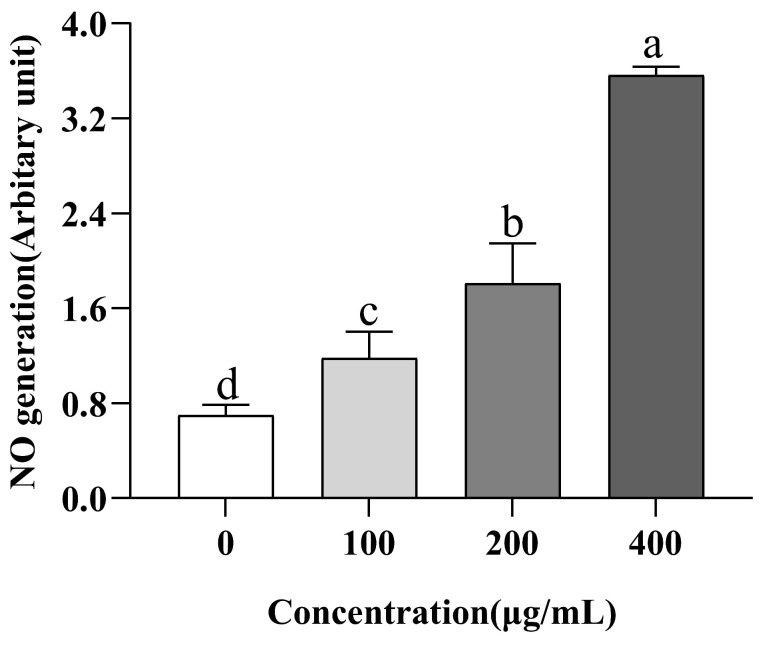
Effect of VNDYLNW on the production of nitric oxide (NO) in EA.hy926 cells. Different letters above the column indicate a significant difference (*p* < 0.05).

NO is a messenger molecular medium that provides multiple functions in biological tissues [52]. Previous studies have shown that the production of NO is one of the potential mechanisms for the prevention and treatment of hypertension [15]. Inhibiting ACE can prevent vasodilator breakdown and directly stimulate endothelial cells to produce NO [16]. We cultured EA.hy926 cells in the medium and evaluated the intracellular NO in order to see how VW-7 affected the generation of NO in these cells. VW-7 significantly increased NO secretion, as demonstrated in Figure 10, when compared to the group receiving no treatment (*p* < 0.05), and the production of NO increased with the increase in peptide concentration. When the concentration of VW-7 was 400 µg/mL, the production of NO was 3.02 times that of 100 µg/mL. There were also a large number of reports in previous studies on ACE inhibitory peptides promoting the production of NO in human endothelial cells. For example, Zhao et al. [51] found that four kinds of antihypertensive peptides significantly promoted the production of NO in HUVEC in the study of antihypertensive peptides in Antarctic Krill. Oh et al. [53] found in their study of antihypertensive peptides in Olive Flounder Surimi that the production of NO in cells was significantly increased after treatment with 25–100 µg/mL WYK and VASVI.

## 4. Conclusions 

This study purified and identified a new ACE inhibitory peptide (VW-7) from *Torreya grandis* meal protein, with an IC_50_ value of 205.98 µM. VW-7 was proven to be a mixed inhibitor. Molecular docking showed that the hydrogen bond interaction with the HEXXH zinc-binding motif (His383) and Zn^2+^ of ACE played an important role in the ACE inhibitory activity of VW-7. In addition, in the in vitro gastrointestinal digestion experiment, VW-7 constantly maintained the same activity and structure, demonstrating high anti-gastrointestinal digestion stability. VW-7, at low concentrations (400 µg/mL), has no cytotoxicity in EA.hy926 cells and can significantly raise the intracellular NO level. Our study confirmed that *Torreya grandis* meal protein can be used as the starting material for the preparation of ACE inhibitory peptides, and VW-7 can be regarded as a promising candidate for application as an antihypertensive agent in functional foods.

## Figures and Tables

**Table 1:** Column elution program.

Time (Min)	A (%)	B (%)
0	95	5
25	35	65
30	5	95
35	5	95
40	95	5

**Table 2:** Peptide bioactivity prediction and molecular docking results.

Peptide Sequence	Binding Energy (kcal/mol)	PeptideRanker Score	Toxicity Prediction
FFFN	−9.6	0.99	Non-Toxin
YTKLEPR	−8.4	0.12	Non-Toxin
DYLYH	−9	0.42	Non-Toxin
FDKLLSPR	−9	0.58	Non-Toxin
KALGAP	−9.1	0.32	Non-Toxin
FFDK	−8.6	0.89	Non-Toxin
YSSAL	−8.7	0.32	Non-Toxin
YSTAL	−9	0.31	Non-Toxin
FFDLK	−8.8	0.87	Non-Toxin
VVPF	−8.7	0.58	Non-Toxin
LLSPR	−7.7	0.45	Non-Toxin
VYLQ	−8.5	0.12	Non-Toxin
QLFVK	−8.3	0.32	Non-Toxin
WMLV	−8.5	0.84	Non-Toxin
FDKL	−8.4	0.67	Non-Toxin
LLSSPR	−7.9	0.41	Non-Toxin
LLAA	−7	0.25	Non-Toxin
FFNVN	−9.2	0.69	Non-Toxin
LRLL	−7.3	0.57	Non-Toxin
VNDYLYH	−9.7	0.34	Non-Toxin
VGYL	−8	0.42	Non-Toxin
SVPNWESH	−9.4	0.34	Non-Toxin
TYLY	−9	0.29	Non-Toxin
YTKLERP	−8.6	0.11	Non-Toxin
GHPLVQ	−8.5	0.25	Non-Toxin
AVVHY	−8.8	0.1	Non-Toxin
GYGL	−8.5	0.79	Non-Toxin
MLPH	−7.5	0.72	Non-Toxin
YTKLEP	−9	0.1	Non-Toxin
FDLK	−7.7	0.58	Non-Toxin
NRNPPSLL	−8.8	0.57	Non-Toxin
YLRPN	−8.7	0.48	Non-Toxin
AYRDF	−10.5	0.79	Non-Toxin
SFMLPH	−8.3	0.88	Non-Toxin
LHNPNPY	−9.6	0.45	Non-Toxin
LSLF	−7.2	0.77	Non-Toxin
VNDYLNW	−10	0.6	Non-Toxin

**Table 3:** Vmax and Km of VNDYLNW at different concentrations.

Parameter	0 mg/mL	0.1 mg/mL	0.4 mg/mL
Vmax (μg/mL·min)	1.12	0.76	0.36
Km (mM)	5.21	6.14	16.79

## Data Availability

The data presented in this study are available on request from the corresponding author.
